# Ultrasound-Guided Puncture Pneumoperitoneum for Laparoscopy. Pilot Study of a New Technique in an Animal Model

**DOI:** 10.1590/0100-6991e-20243789-en

**Published:** 2024-11-07

**Authors:** JENNIFER MELISSA DE OLIVEIRA MARQUES, CAMILA MARIA PINTO FERREIRA VERARDINO, LUIS GUSTAVO CAPOCHIN ROMAGNOLO, ARMANDO GERALDO FRANCHINI MELANI, RODRIGO CHAVES RIBEIRO

**Affiliations:** 1 - Faculdade de Ciências da Saúde de Barretos Dr. Paulo Prata - FACISB, Medicina - Barretos - SP - Brasil; 2 - IRCAD América Latina - Unidade de Barretos - Barretos - SP - Brasil; 3 - Hospital de Câncer de Barretos, Departamento de Digestivo Baixo - Barretos - SP - Brasil; 4 - Hospital Infantojuvenil de Barretos. Hospital de Câncer de Barretos, Departamento de Cirurgia Pediátrica - Barretos - SP - Brasil

**Keywords:** Pneumoperitoneum, Laparoscopy, Ultrasonography, Pneumoperitônio, Laparoscopia, Ultrassonografia

## Abstract

**Introduction:**

All forms of access to the peritoneal cavity in laparoscopy could damage intra-abdominal structures. Currently, ultrasound (USG) is being used in several procedures to guide needles: breast biopsy, central venous access puncture, anesthetic nerve blocks, etc. Therefore, this research seeks to verify the feasibility and viability of performing pneumoperitoneum using USG-guided puncture in a pilot study using a porcine model.

**Methods:**

The cross-sectional study was carried out with a sample of 10 anesthetized sows in the IRCAD-América Latina Barretos Unit laboratory. The experiment consisted of an abdominal puncture guided by USG with a linear transducer to create the pneumoperitoneum. After the puncture, the drop test was performed, and CO2 was insufflated into the cavity. Subsequently, a 10mm trocar was introduced to insert the optic. The parameters from the USG were the thickness of the abdominal wall layers, intraperitoneal needle measurement, drop test, and the presence of complications.

**Results:**

The average measurement of the layers was 0.45 centimeters of subcutaneous tissue, 0.67 centimeters of muscle, and 0.15 centimeters of peritoneum. The mean measurement of the intraperitoneal needle was 1.17cm. Furthermore, the drop test was positive in 100% of cases, and there was no bleeding or lesions on any attempt.

**Conclusion:**

Ultrasound-guided pneumoperitoneum is feasible and safe in the porcine model. The subcutaneous, muscular, and peritoneum layers are identifiable and measurable in this model. Subsequent studies are necessary to verify the importance of this new procedure.

## RESUMO

**Introdução:**

Todas as formas de acesso a cavidade peritoneal na laparoscopia possuem riscos de lesionar as estruturas intra-abdominais. Atualmente, a ultrassonografia (USG) está sendo utilizada em diversos procedimentos para direcionar algum tipo de punção: biópsia de mama, acesso venoso central, bloqueios anestésicos de nervos etc. Diante disso, esta pesquisa busca verificar a factibilidade e viabilidade da realização do pneumoperitônio por punção guiada por USG, em um estudo piloto em modelo porcino.

**Métodos:**

O estudo experimental foi feito com uma amostra de 10 porcas anestesiadas, no laboratório do IRCAD-América Latina Unidade de Barretos. O experimento consistiu na punção abdominal guiado por USG com transdutor linear para confecção do pneumoperitônio. Após a punção, foi realizado o teste da gota e insuflado CO2 na cavidade, posteriormente, um trocarte de 10mm foi introduzido para inserção da óptica. Os parâmetros a partir do USG foram: as espessuras das camadas da parede abdominal; medida da agulha intraperitoneal; teste da gota; e presença de complicações.

**Resultados:**

A mensuração da média das camadas foi de 0,45 centímetros (cm) de subcutâneo, 0,67cm de muscular e 0,15cm de peritônio. A média da medida da agulha intraperitoneal foi de 1,17cm. Ademais, o teste da gota foi positivo em 100% dos casos e não houve sangramento ou lesões em nenhuma tentativa.

**Conclusão:**

É factível e seguro a realização de pneumoperitônio guiado por ultrassonografia no modelo porcino. As camadas subcutâneas, muscular e peritônio são identificáveis e mensuráveis no modelo. Estudos subsequentes são necessários para verificar a importância deste novo procedimento.

## INTRODUCTION

Laparoscopic surgeries have become a routine in general surgery and in several other surgical specialties (gynecological, urological, pediatric, coloproctological, bariatric). In recent years, new equipment has been developed that has allowed increasingly complex surgeries through minimally invasive access, such as tweezers, needle holders, articulated scissors, sealers, staplers, high-resolution optical systems, fluorescence, three-dimensional imaging, and the advent of robotic surgery^1^. Despite these technological advances, there are still challenges faced in performing some stages of minimally invasive procedures, such as access to the peritoneal cavity and instillation of pneumoperitoneum^2^. Complications attributed to laparoscopy, although rare, are potentially severe, one third of them correspond to injuries through the access to the abdominal cavity, such as perforation of intestinal loops, vessels, and adjacent organs^1^ ^-^ ^3^. 

Entry into the abdominal compartment can be performed in three main ways: by the closed technique with the Veress needle, by direct insertion of trocars, or by the open technique, with incision by planes of the abdominal wall^4^. In most abdominal surgery services, access is made blindly with the Veress needle, which is also the preferred method by gynecologists. However, as in other options for performing pneumoperitoneum, there is a risk of vascular injury or viscera perforation^4^ ^-^ ^6^. Over the years, some safety checks have been created for this type of entry, such as the double click of the Veress needle, the aspiration test, the drop of suspended saline solution, the wheezing sound, and the syringe sound. Still, even they could not reduce the occurrence of complications in obtaining pneumoperitoneum^7^ ^-^ ^9^. In the method of peritoneal access with passage of the trocar under direct vision, with traction of the abdominal skin, there is no need to inflate the cavity before introducing the first trocar, which can reduce the risks of gas embolism due to high intra-abdominal pressures, but the risks of lesions of vessels and bowel loops are not lower than in other strategies^10^ ^-^ ^12^. The open technique, on the other hand, consists of dissection of the abdominal wall layers, plane by plane, and although it appears to have a reduced risk of vascular injury, its advantages over other entry techniques are not proven, in addition to being associated with a higher rate of wound infection, intestinal perforations, and air leaks during the procedure^13^. Therefore, using any of the techniques will present advantages and disadvantages in specific subgroups of patients or when performed by different professionals with different skills. For these reasons, there are several guidelines for the prevention of complications related to the abdominal cavity access approaches, but the gold standard is still under debate in the literature^2^ ^,^ ^5^.

In recent decades, ultrasonography (USG) has been used by non-radiologists to perform various procedures, such as long or short-term central venous accesses, peripheral accesses in dehydrated patients, guided biopsies, nerve blocks (in anesthesiology or as a treatment for chronic pain), initial evaluation of polytrauma patients, hemodynamic evaluation in intensive care, ablation of solid tumors, and guided paracentesis^14^. The use of ultrasound in central venous access, for example, provides better visualization of the anatomical structures of the cervical region, such as muscles, arteries, and veins, thus allowing a decrease in the occurrence of severe complications such as hemothorax and pneumothorax^14^ ^-^ ^16^. In breast biopsies, USG improves the recognition of the breast structures, in addition to adequately limiting the biopsy^17^ ^,^ ^18^. In anesthesiology and physiatry, USG-guided nerve blockade allows the deposition of the anesthetic exactly around the nerves, thus obtaining a more effective blockade with lower latency, less dependence on anatomical references as in the blind procedure, reduction of the chances of harming adjacent structures, use of smaller anesthetic volumes, and greater safety^19^ ^-^ ^22^. In critically ill patients, USG can be used to measure echogenicity and thickness of the muscle layers, associating it with decreased muscle strength, which suggests that echogenicity could aid in prognosis, while the patient is still unable to perform tests with voluntary movement^23^ ^-^ ^25^. USG-guided puncture can also be used for abdominal procedures, such as ablation of liver tumors^26^ ^,^ ^27^ and draining of ascitic fluid and intracavitary collections^28^, to obtain more assertiveness and fewer complications. However, there is no description of ultrasound-guided puncture for pneumoperitoneum in laparoscopic procedures. Abdominal ultrasonography using a linear transducer, such as the one used for abdominal block (“TAP-Block”), in addition to blood vessels and intra-abdominal organs, can recognize all the layers of the abdominal wall, as well as differentiate the types of tissues in the region^23^ ^,^ ^29^ ^,^ ^30^: subcutaneous cellular tissue, aponeurosis of the rectus abdominis and external oblique muscles, and the external oblique, internal oblique, transverse, and rectus abdominis muscles^31^ ^-^ ^33^. In addition to an accurate verification of the anatomical structures in procedures such as central venous access, breast biopsy, and nerve block, USG can show the path of the needle crossing the muscle layers^14^ ^-^ ^22^.

Therefore, this study aims to verify the feasibility and safety of performing ultrasound-guided puncture pneumoperitoneum in an animal model.

## METHODS

We conducted an experimental study in an animal model to verify the feasibility and safety of pneumoperitoneum by ultrasound-guided puncture. The research was developed at the Institute for Training in Minimally Invasive Surgeries - IRCAD, Barretos’ unit, where courses are offered for various medical specialties in minimally invasive surgeries and robotics. The laboratory’s structure, equipment, and veterinarians were used to carry out the study tests in September 2022.

The models for testing were ten female pigs of the Large White breed from suppliers registered to IRCAD, weighing between 20 and 30 kilograms (kg), aged between six and eight months. The animals did not have any alteration of the abdominal cavity or previous procedures that could interfere with the study. The research sample was small because it is an initial pilot study (convenience sample), without sample calculation. The animals were anesthetized by seasoned veterinarians with the combination of Tefazol (5mg/kg, Intramuscular), Xylazine (1.5mg/kg), and Atropine administered for endotracheal intubation, followed by Isoflurane (2%). During anesthesia, the pigs were monitored with a cardioscope, oximetry, and maintenance of intravenous hydration with saline. These animals were destined for the courses that IRCAD offers, and the experiment was carried out minutes before the start of the practical courses. In other words, animals that would already be anesthetized and submitted to pneumoperitoneum were used to perform surgical procedures in some courses of minimally invasive surgery at the IRCAD. After the end of the course training, all pigs went through the euthanasia process, following the IRCAD standards with a lethal dose of potassium chloride (2mEq/kg KCl), and were later cremated. 

All procedures performed were in accordance with the ethical standards of animal research, and the Ethics Committee on the Use of Animals (CEUA) approved this study (process number 7156260722). In this article there are no human trials, so the informed consent form did not apply.

### Technique Description

After administering anesthesia to the pigs, the surgical drapes, the laparoscopy tower, and the ultrasound machine were placed to the left of the animal. Initially, a mapping of the animal’s abdominal wall was done to identify the muscle part, the subcutaneous tissue, and the peritoneum. For this, we tested some ultrasound devices, and the one that provided better visualization was the GE’s Vivid and linear transducer. After adjusting the depth and calibrating the device, the abdominal wall was visualized through a transverse window, where it was possible to identify the union of the lateral muscles of the abdomen (transverse, external oblique, internal oblique, and rectus abdominis), subcutaneous tissue, peritoneum, and viscera. The introduction of the needle was standardized for all animals, with a puncture in the intermammillary line at the level of the umbilical scar, parallel to the transducer, to be visualized by ultrasonography as a hyperechoic linear image that crosses the layers of the abdominal wall, i.e., the so-called “in plane” puncture. We estimated the portion of the needle inside the abdomen by subtracting, from the total size of the Abocath 14 needle, which is 4.5cm, the length of the needle outside the skin and the measurement of the needle inside the abdominal wall. The latter was estimated using the wall thickness, considering the puncture angle of 45 degrees, and applying these measurements to the Pythagorean Theorem. After the needle penetrated the cavity, we performed the drop test with an infusion of 5ml of 0.9% saline solution, whose descent indicates a change in pressure, suggesting introduction into the intraperitoneal space. With the positive test, carbon dioxide was instilled with a Storz inflator, with a flow rate of 5l/min and a maximum pressure of 5mmHg. After reaching the target pressure, the incision was enlarged to pass a 10-mm trocar. This insertion was in the same path as the needle, and the USG was used to visualize the insertion. After the trocar, optics were introduced to verify whether the pneumoperitoneum was effective and whether there was any injury to intra-abdominal organs or blood vessels.

### Collected Variables

The parameters evaluated by ultrasonography were subcutaneous thickness, lateral muscle thickness, peritoneal thickness, and the of length the needle penetrated the peritoneal cavity. The drop test was also a parameter analyzed: positive, doubtful, or negative. Another was CO2 inflation: adequate, high-pressure peaks, and others. Moreover, intraperitoneal visualization with optics was used to identify complications such as viscera lesions and bleeding.

The sample was characterized through frequency tables for categorical variables and measures of central tendency (mean, median) and dispersion (standard deviation) for quantitative variables.

## RESULTS

**Figure 1:**
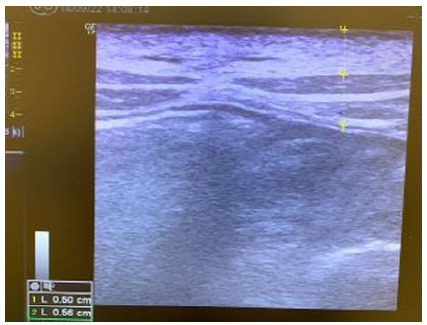
Measurement of the abdominal wall layers by ultrasound.

Initially, we tested which of the USG devices and which needle would offer better visualization, opting for GE’s USG Vivid and the Abocath for the puncture. In the first two pigs, we used an Abocath 16, but there was difficulty in obtaining adequate pressure for pneumoperitoneum formation, so we switched to Abocath 14 in the subsequent animals, acquiring effective pneumoperitoneum in a timely manner. In all animals, it was possible to identify the subcutaneous tissue, muscle layer (oblique, transverse, and rectus abdominis muscles), and peritoneum (Figure 1) with the USG device. We then proceeded to measure the layers. The averages of each layer were 0.45 centimeters (cm) for the subcutaneous, 0.67cm for the muscle, and 0.15cm for the peritoneum. Table 1 and Figure 1 show the measurement of the abdominal wall layers of the porcine models.

**Table 1:** Measurement of the abdominal wall layers.

	Subcutaneous thickness	Rectus abdominis muscles thickness	Peritoneum thickness	Total thickness	Diagonal thickness at 45 degrees
Pig 1	0,34	0,63	0,20	1,17	1,65
Pig 2	0,50	0,56	0,06	1,12	1,58
Pig 3	0,35	0,44	0,05	0,84	1,19
Pig 4	0,34	0,63	0,27	1,24	1,75
Pig 5	0,62	0,77	0,16	1,55	2,19
Pig 6	0,53	0,56	0,20	1,29	1,82
Pig 7	0,54	0,72	0,22	1,48	2,09
Pig 8	0,51	0,90	0,09	1,50	2,12
Pig 9	0,39	0,62	0,11	1,12	1,58
Pig 10	0,41	0,90	0,10	1,41	1,99
Average	0,45	0,67	0,15	1,27	1,80
Median	0,46	0,63	0,14	1,27	1,79
Standard deviation	0,10	0,15	0,07	0,22	0,31

**Figure 2:**
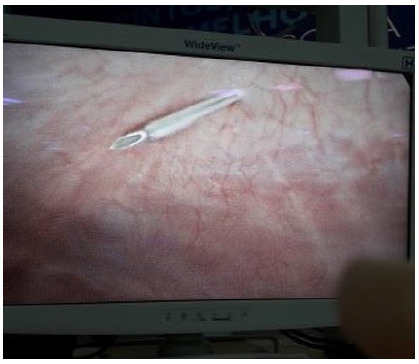
Introduction of the Abocath 14 into the abdominal cavity.

After the in-plane introduction of the Abocath, we measured the extent to which the needle penetrated the abdominal cavity (Figure 2). The mean needle introduction length was 2.97cm, and the mean (?), 1.17cm (Table 2).

**Table 2:** Needle insertion measurements.

	Diagonal thickness at 45º	External needle length	Needle penetration length	Needle into cavity
Pig 1	1,65	2,00	2,50	0,85
Pig 2	1,58	1,90	2,60	1,02
Pig 3	1,19	0,80	3,70	2,51
Pig 4	1,75	1,20	3,30	1,55
Pig 5	2,19	1,80	2,70	0,51
Pig 6	1,82	1,60	2,90	1,08
Pig 7	2,09	0,70	3,80	1,71
Pig 8	2,12	0,90	3,60	1,48
Pig 9	1,58	2,50	2,00	0,42
Pig 10	1,99	1,90	2,60	0,61
Average	1,80	1,53	2,97	1,17
Median	1,79	1,70	2,80	1,05
Standard deviation	0,31	0,60	0,60	0,65

**Figure 3:**
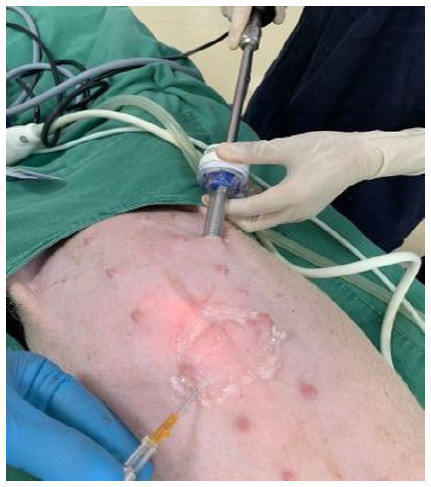
Positioning of the trocar for the introduction of the optics.

**Figure 4:**
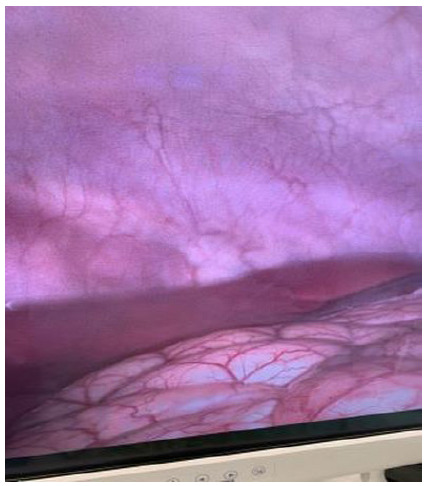
Abdominal cavity of a porcine model without lesions.

Subsequently, we performed the drop test to check if the needle was inside the peritoneal cavity. The test was positive in 100% of the cases. Then, the pneumoperitoneum was instilled with carbon dioxide, and a 10-millimeter trocar was introduced for the optics (Figure 3). When viewing the abdominal cavity with the optics, we found no bleeding or visceral injury in any of the attempts (Figure 4).

## DISCUSSION

Most of the complications related to laparoscopic surgeries occur at the time of entry into the peritoneal cavity for pneumoperitoneum instillation. Such problems are rare but have severe and potentially lethal consequences^11^. Thus, the creation of techniques that minimize these complications during access to the abdominal cavity is essential.

Surgical complications of access to the peritoneal cavity can be divided into early and late. Early bleeding is observed intraoperatively and can result from the puncture of a blood vessel or spleen. In this context, the patient is exposed to several risk situations depending on the severity of the bleeding, such as hypotension, hypoperfusion of vital organs, and cardiorespiratory arrest, the main consequence of intraoperative hemorrhage^34^. Situations like these require not only dexterity from the surgeons in the field, but agility from the entire team involved in the procedure and the resources made available by the hospital. Late complications are identified in the postoperative period, 24 to 48 hours after surgery, and may result from a perforation of an intestinal loop or some vascular injury not perceived during surgery^1^ ^,^ ^3^. Acute abdomen caused by intestinal perforations or hemorrhages requires surgical reapproaches and may require reconstruction of intestinal transit^1^. From this, a new range of complications emerges, such as anastomotic dehiscence, abdominal sepsis, and the need for colostomies, all of which can be worsened further depending on the patient’s comorbidities and pathological history^35^. 

Among the various methods for instilling pneumoperitoneum in laparoscopic surgeries, such as Veress needle access, direct entry with trocars, and the open technique, there are several verification tests to ensure that the instruments are intraperitoneal and avoid complications. However, the use of such tests did not reduce their occurrence^2^. The open technique may seem safer than the others since the incision occurs in layers, but there is no evidence that it reduces visceral and vascular lesions compared with other methods^2^ ^,^ ^3^.

Ultrasonography is used in several puncture procedures to reduce accidents. Despite being operator-dependent, it has grown exponentially in recent years. In addition, training with USG has been introduced even at the most basic levels of medical education, such as undergraduate ones^4^ ^,^ ^5^. This increase in interest in learning how to handle ultrasound devices improves the long-term utilization of this technology in various procedures to reduce complications or evaluate internal organs^6^. For example, ultrasound-guided central venous access uses the ultrasound device to visualize the anatomical structures as the needle penetrates the correct puncture site, thus minimizing the occurrence of pneumothorax and hemothorax^14^ ^-^ ^16^. In USG-guided anesthetic nerve blockade, it is possible to anesthetize the desired nerves much more precisely and to divert important structures, such as blood vessels adjacent to the area to be blocked^7^ ^,^ ^19^ ^-^ ^22^. The same occurs in the ablation of solid abdominal neoplasms with radiofrequency^26^ ^,^ ^27^. Thus, the function of ultrasound in all these procedures is to recognize organs and blood vessels, guide the needle, and, consequently, reduce complications, making these procedures safer.

The present study thus sought to apply the same principles as in the use of USG in the mentioned procedures for the puncture and instillation of pneumoperitoneum in laparoscopic surgeries. As we found no previous description for this same purpose, the proposal was to test it in a porcine model as an initial pilot study. The identification of the layers of the animal’s abdominal wall, as well as the visualization of the needle through a USG device, allowed better control in the performance of the puncture^4^ ^,^ ^5^ ^,^ ^9^ and consequent reduction of complications. Although not addressed in this study, Doppler ultrasonography would be able to identify blood vessels and avoid possible vascular lesions^36^. However, for the use of Doppler in USG, there would be a need for specific habilitation. The purpose of this study is that soon any surgeon will be able to use USG to instill a pneumoperitoneum, as it is used in other procedures and by other specialists to perform central venous access and evaluation of trauma patients, for example. 

The results of this study highlight the benefits of using USG to guide procedures, in addition to being a low-cost, easily accessible proposal that has been introduced in several other areas of medicine so that its management is more widespread. There are some limitations regarding the sampling used. Although the porcine model is very well established in the literature, having been used in several scientific studies in the surgical area, there are considerable differences between the characteristics of the skin of this animal and that of humans, which may confer divergences at the time of puncture. Furthermore, the small sample cannot prove that this pneumoperitoneum technique is superior to the ones already established. In addition, it is not possible to infer that this method will reduce complications of access to the abdominal cavity, such as intestinal lesions. We are waiting for the sample to be expanded in subsequent work and more prospective studies on the subject to better understand and define its indication as a prevention of complications in establishing pneumoperitoneum. The study considers the hypothesis that ultrasound-guided pneumoperitoneum will have an impact on special situations, such as obese patients, patients with previous abdominal surgeries, and pregnant women. It is also an initial pilot project for this line of research.

## CONCLUSION

Ultrasound-guided pneumoperitoneum in a porcine model is feasible and safe. The subcutaneous and muscle layers and the peritoneum are identifiable and measurable in the model. Subsequent studies are needed to verify the importance of this new procedure.
